# Trophic divergence of Lake Kivu cichlid fishes along a pelagic versus littoral habitat axis

**DOI:** 10.1002/ece3.7117

**Published:** 2021-01-27

**Authors:** Philippe S. Munyandamutsa, Wilson L. Jere, Daud Kassam, Austin Mtethiwa

**Affiliations:** ^1^ Africa Centre of Excellence in Aquaculture and Fisheries Science Department of Aquaculture and Fisheries Science Bunda College Lilongwe University of Agriculture and Natural Resources Lilongwe Centre Malawi; ^2^ Department of Animal Production College of Agriculture, Animal Sciences and Veterinary Medicine University of Rwanda KK 737 Musanze North Rwanda

**Keywords:** haplochromines, kinematic transmission, Lake Kivu, local adaptation, trophic morphology

## Abstract

Local adaptation to the littoral and pelagic zones in two cichlid haplochromine fish species from Lake Kivu was investigated using morphometrics. Cranial variation and inferred jaw mechanics in both sexes of the two species across the two habitat types were quantified and compared. Comparisons of littoral versus pelagic populations revealed habitat‐specific differences in the shape of the feeding apparatus. Also, kinematic transmission of the anterior jaw four‐bar linkage that promotes greater jaw protrusion was higher in the pelagic zone than in the littoral zone for both species. Inferred bite force was likewise higher in pelagic zone fish. There were also sex‐specific differences in craniofacial morphology as males exhibited longer heads than females in both habitats. As has been described for other cichlids in the East African Great Lakes, local adaptation to trophic resources in the littoral and pelagic habitats characterizes these two Lake Kivu cichlids. Similar studies involving other types of the Lake Kivu fishes are recommended to test the evidence of the observed trophic patterns and their genetic basis of divergences.

## Graphical abstract

Comparisons of littoral versus pelagic Lake Kivu haplochromine species revealed habitat‐specific differences in the shape of the feeding apparatus.

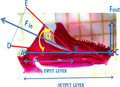

## 1 INTRODUCTION

East African cichlids are well‐known for their phenotypic divergence over short time scales and across small geographic distances (Koblmüller et al., 2019; Marques et al., 2019; Rajkov et al., 2018; Schneider & Meyer, 2017). Adaptive evolution in these fishes commonly results from habitat divergence and/or trophic specialization (Chukwuka et al., 2019; Clabaut et al., 2007; Colombo et al., 2016; Gunter & Meyer, 2014; Muschick et al., 2012; Rajkov et al., 2018). Many adaptive traits such as body size and shape are tightly linked in cichlids and other vertebrates to both the physical environment and resource use (Duarte et al., 2016; Hulsey et al., 2013; Kassam et al., 2003, 2007; Theis et al., 2017). However, cichlids are best‐known for their extensive adaptive divergence in trophic structure and jaw mechanics (Holzman et al., 2012; Hulsey & Garcia de León, 2005; Muschick et al., 2014; Wainwright et al., 2001). This study examines whether several trophic traits that are known to diverge predictably in other fishes inhabiting different lake habitats show divergence in two species of haplochromine cichlids from Lake Kivu: *Haplochromis insidiae* (Snoeks, 1994) and *Haplochromis. kamiranzovu* (Snoeks, 1984).

Fish jaw muscles and bones often display predictable morphological divergence in littoral versus pelagic habitats. Many of these changes along the littoral to pelagic axis in cichlids involve the size and shape of the preorbital region of the skull (Amaral & Johnston, 2012; Gerry et al., ; Parsons et al., 2011, 2015). Skeletal elements that include the opercular, orbital, and suspensorial bones commonly differ between littoral and pelagic fish (Bartels et al., 2012; Jones et al., 2013; Lucia et al., 2013; Muschick et al., 2012; Olsson & Eklöv, 2005). The heads of fishes are also densely packed with functional systems that contribute to feeding abilities in different environments. Suction feeding is more common in pelagic habitats while biting is more common in littoral habitats (Adams et al., 1998; Barel, 1983; Conith et al., 2018; Gerking, 1994; Huckins, 1997; Tkint et al., 2012; Wainwright, 1996). For example, the length of the ascending arm of the premaxilla can influence bite force and also the maximum distance that fish protrude their jaws (Hulsey, Hollingsworth et al., 2010; Hulsey, Mims et al., 2010; Witte, 1983). Additionally, traits that can be modeled as simple lever systems such as the lower jaw and the anterior jaw four‐bar linkage are often involved in cichlid trophic divergence (Holzman et al., 2012; Hulsey & Garcia de León, 2005; Hulsey, Hollingsworth et al., 2010; Hulsey, Mims et al., 2010). Quantifying these traits in fishes from both the littoral and pelagic habitats of Lake Kivu would allow us to test whether cichlids diverge along the pelagic versus littoral habitat axis according to the general patterns observed in other fishes.

Lake Kivu is located between Rwanda and the Democratic Republic of Congo (DRC). In Rwanda, the water surface area of Lake Kivu covers 790 km^2^ with a maximum depth of approximately 489 m. The lake is freshwater, meromictic, and oxygenated waters limited to 60 m depths and permanently separated from deep waters by salinity gradients (Degens et al., 1973; Isumbisho et al., 2006). The littoral area is defined for this study as ranging consistently from the water surface to 50 meters deep; the reference was made to the hydroacoustic survey (Snoeks et al., 2012). Lake Kivu is the smallest of the East African Great Lakes (Schmid et al., 2005). It is connected to Tanganyika via the Rusizi River (Haberyan & Hecky, 1987). The ecomorphology of two small species of haplochromine cichlids (50–100 mm SL) was investigated.

The sex of Lake Kivu cichlids could also influence their trophic divergence (Hendry et al., 2006; Herler et al., 2010; McGee & Wainwright, 2013; Shine, 1989). Sex‐specific energetic or nutritive requirements associated with producing offspring in these mouthbrooding fish might commonly lead to different trophic morphologies (Belovsky, 1978; Slatkin, 1984). Additionally, in organisms with substantial parental care such as haplochromine cichlids, differences in responsibilities to offspring might commonly lead to sex‐specific trophic habits (Wheatley, 1972). Furthermore, in adaptively diverging populations, ecologically relevant traits could be linked to mating preferences (Qvarnstrom & Bailey, 2009), and craniofacial selection for maternal mouthbrooding can act in opposition to selection for better feeding performance. For instance, in the *Herichthys minckleyi* of Cuatro Ciénegas cichlids there exists a phenotype association between sex and a number of traits that influence trophic divergence (Hulsey et al., 2015). Sexual shape dimorphism might be expected to be related to divergence along the littoral versus pelagic habitat in Lake Kivu cichlids. Only female haplochromines are mouth‐brooders. They are endemic to the Lake Kivu and typically planktivorous (Snoeks, 1994). The age of divergence between the two haplochromines species is still unknown.

Lake Kivu contains quite distinct littoral and pelagic habitats that could influence cichlid trophic divergence. Although the littoral zone constitutes only 10% of Lake Kivu's surface area, its ecological distinctiveness is suggested by the many fish species that are confined to this habitat (Snoeks, 1994). The littoral zone of Lake Kivu has a benthic substrate composed of rocks, macrophytes, mud, as well as sand, and a relatively rich macro‐invertebrate community inhabits this varied substrate (Verbeke, 1957). However, phytoplankton, diatoms, and rotifers are most abundant in the pelagic zone where densities of these organisms are 15–50 times higher than in the littoral zone (Isumbisho et al., 2006; Sarmento et al., 2006). Importantly, in the pelagic zone, oxygen concentrations decrease rapidly below 50 m and becomes effectively zero at 70 m due to the approximately 60 km^3^ of methane dissolved in the permanently stratified waters of Lake Kivu (Schmid et al., 2005; Tietze, 1981). This anaerobic environment effectively excludes pelagic zone fish from ever feeding from the substrate. The distinctiveness of the littoral versus pelagic habitats in Lake Kivu could likely structure fish trophic divergence.

*Haplochromis insidiae* and *H. kamiranzovu* live in both the pelagic and littoral environment. We do not know yet how closely related these species are to be able to consider phylogenetically independent populations. However, they belong to the same genus. It is unknown if they are genetically isolated.

These haplochromines fish species were examined to answer the following questions:

1. Are there species‐specific differences in musculoskeletal shape between pelagic and littoral fish?
2. Are there species‐specific musculoskeletal shape differences between the sexes?
3. Are the differences in trophic morphology within each species similar between the two species?

To examine the above questions, the morphological variations in cranial musculoskeletal traits that influence feeding performance were compared.

## 2 MATERIALS AND METHODS

### 2.1 Study area, specimen collection, and dissection

**FIGURE 1:**
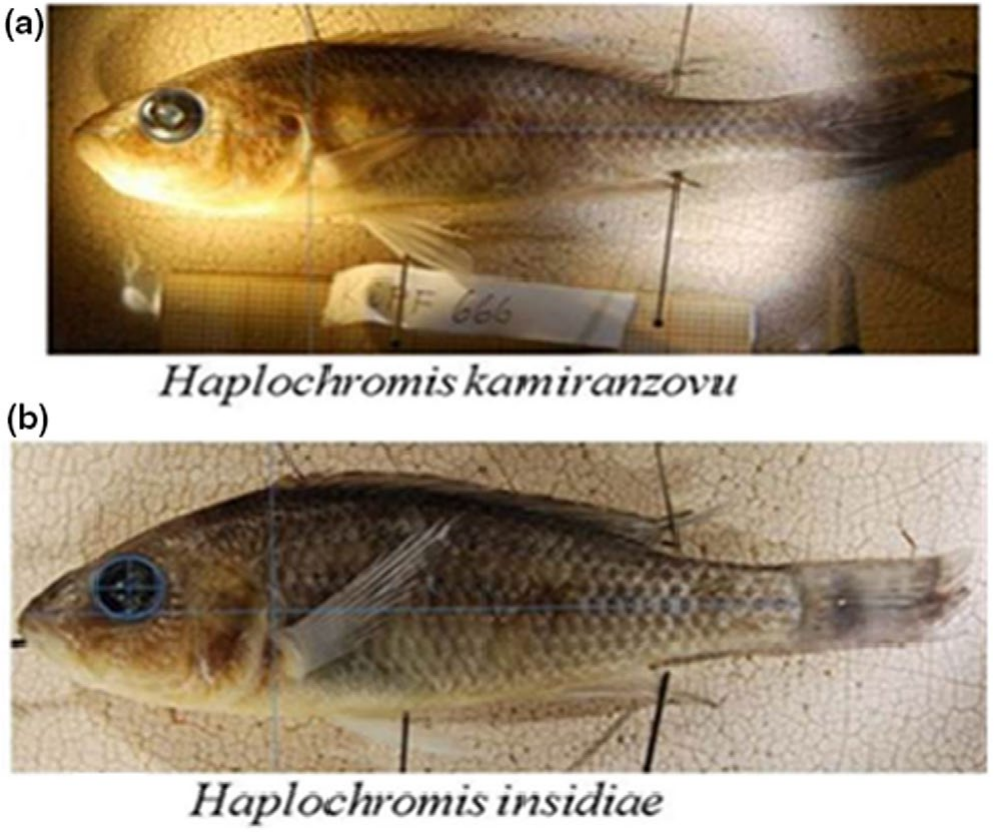
Haplochromines fish species studied

**FIGURE 2:**
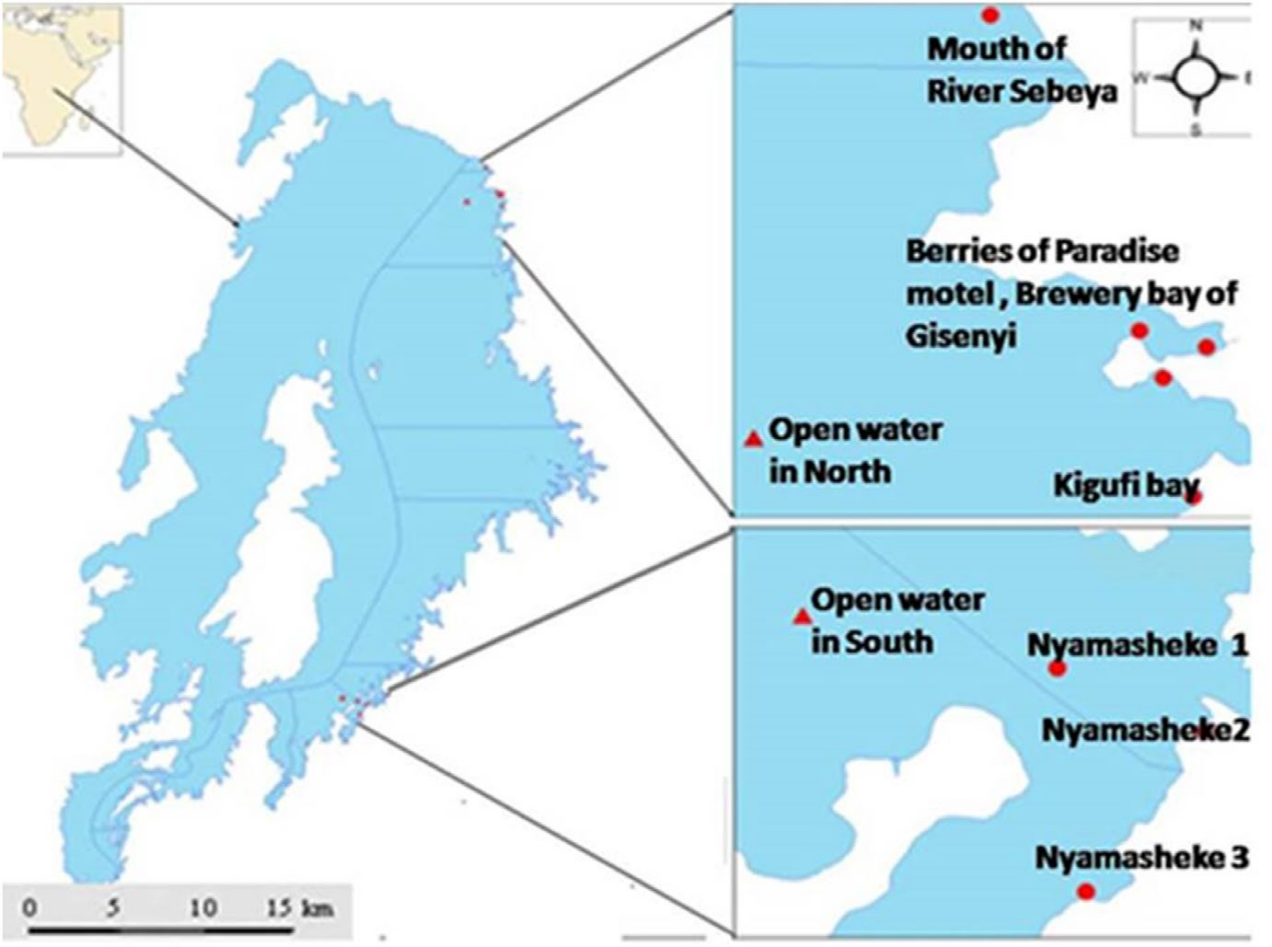
Map of Lake Kivu and fish sampling locations. Dots represent littoral sampling locations, triangles represent pelagic sampling locations. Blue lines represent the expansion of the sampling area; northern and southern regions. GPS coordinates of Nyamasheke 1 are closer to an island of less than 40 meters depth. Other GPS coordinates of Nyamasheke sampling locations are closer to the coastal zone. In the littoral north, two berries of Paradise Motel and one brewery bay of Gisenyi counted also for our fish sampling

Adult fish (Figure 1) were caught in littoral and pelagic zones of northern and southern regions of Lake Kivu using 15 m by 1 m gillnets made from monofilament nylon (10 mm mesh size). In the north, the fish were sampled from the Brewery bay of Gisenyi, Berries of Paradise motel, Kigufi bay and Mouth of Sebeya River. After several unfruitful sampling of the targeted species in many places along the southern shore, the two haplochromines species were found only in Nyamasheke. Therefore, sampling was done at Nyamasheke 1, Nyamasheke 2, and Nyamasheke 3. In addition, one sampling was done in the open waters of the northern part of the lake and another sampling was done in the open waters of the southern part (Figure 2). A total of 95 individuals of 2 fish species (*H. insidiae* and *H. kamiranzovu*) were sampled (Figure 2; Table 1).

**TABLE 1:** Number of fish specimens cleared and stained was grouped per zone of the sampling site in the lake and is reported in the following table

Habitat	♂ I/♂K	♀ I/ ♀ K	Total
Littoral	10/13	11/13	21/26
Pelagic	11/10	13/14	24/24
Total	21/23	24/27	45/50

Note

♂, male; ♀, female; I, *Haplochromis insidiae*; K, *Haplochromis kamiranzovu*.

Fish were taxonomically identified using fin, body, and tooth traits (Snoeks, 1994). *Haplochromis kamiranzovu* has a more elongated caudal peduncle and a smaller body depth compared to *H. insidiae*. In contrast to other haplochromines endemic to Lake Kivu, these two species tend to have a higher number of gill rakers and achieve larger body sizes (Snoeks, 1994). They also have distinct tooth shapes (Snoeks, 1994), with the major tooth cusp of *H. kamiranzovu* being relatively large and pointed while the major tooth cusp of *H. insidiae* being less curved. Individual sexes were determined by examination of gonads.

Initially, the skin was removed from the head of fish to allow measurements of the three adductor mandibular (A1, A2, and A3). The A3 adductor mandibular is internal and cannot be seen.

This complex of muscles adducts the jaws and powers oral jaw biting (Anker, 1978; Hulsey et al., 2007; Westneat, 1995a, 1995b, 2003, 2004).

### 2.2 Geometric morphometrics

Subsequently, specimens were cleared and stained following the protocol of Taylor and Van Dyke (1985). Clearing and staining was done in nine consecutive steps: (1) dissection and removal of skin, (2) removal of the gastrointestinal track and gonads, (3) dehydration in 95% ethanol, (4) placement of the fish into Alcian blue staining for cartilage, (5) neutralization, (6) bleaching the specimens in 15% hydrogen peroxide and 85% potassium hydroxide solution, (clearing step 1), (7) staining for bone in Alizarin red solution, (8) placement of the specimens into trypsin solution (clearing step 2), and (9) putting the specimens into glycerine.

During dissections, a photograph of the head, muscles, and the ligamentous insertions of the adductor mandibular was taken. Each muscle was dissected then, weighed on an electronic balance (Sartorius BP 121S) to the nearest 0.1 mg, and later used for physiological cross section calculation.

**FIGURE 3:**
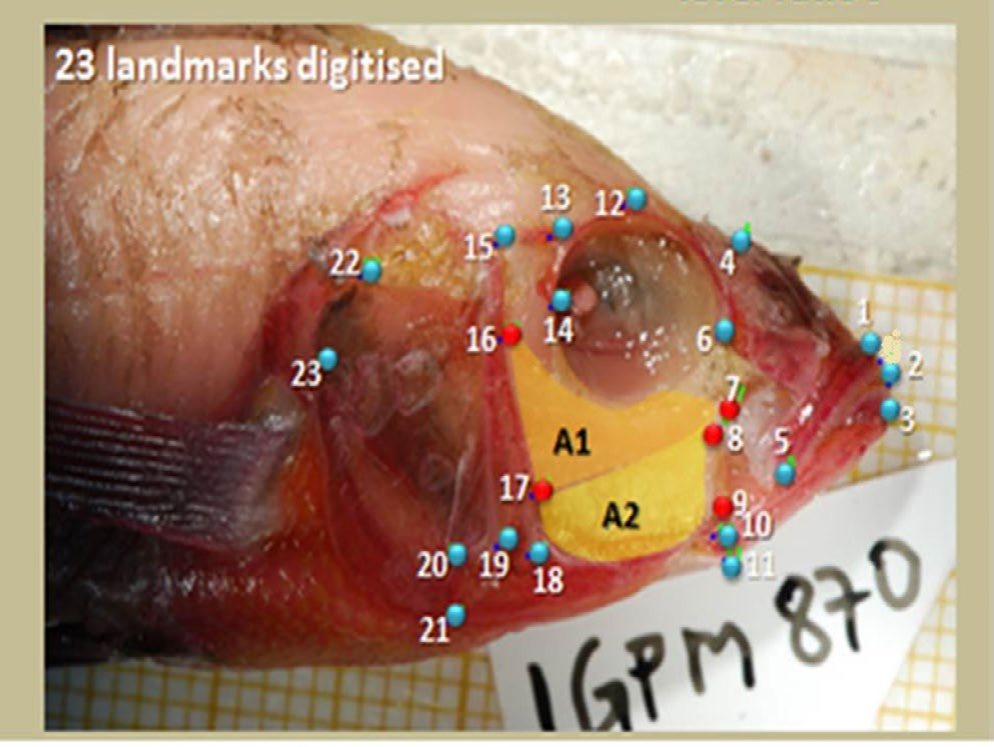
Landmarks used to capture the head shape variation of the two species dissected. Red and blue dots indicate muscular, skeletal landmarks, musculoskeletal shape, muscle size, insertion angles, and lever ratio's. (1) Rostral tip of the premaxilla; (2) Tip of the anterior most tooth on the premaxilla; (3) Anterior most tip of the lower jaw; (4) Posterior tip of the ascending process of the premaxilla; (5) Posterior end point of the dentigerous process of the premaxilla; (6) Most anterior‐ventral point of the eye socket; (7) Tip of the anterior most point of the A1 division of the adductor mandibulae; (8) Tip of the anterior most point of the A2 division of the adductor mandibulae; (9) Most antero‐ventral point of the A2 division of the adductor mandibulae; (10) Lower jaw joint; (11) Retroarticular process; (12) Dorsal supraorbital lateral line foramen (nlf3); (13) Posterior‐dorsal supraorbital neurocranial lateral line foramen (nlf4); (14) Most posterior‐ventral point of the bony eye‐socket; (15) Dorsal preopercular lateral line foramen (nlf5); (16) Most dorsal point on the origin of the A1 division of the adductor mandibulae jaw closing muscle on the preopercular; (17) Most dorsal point on the origin of the A2 division of the adductor mandibulae jaw closing muscle on the preopercular; (18) Ventral preopercular lateral line foramina (slf4); (19) Ventral contact point of the subopercle and preopercle; (20) Ventral tip of the opercle; (21) Ventral intersection point of the subopercle and interopercle; (22) Posterior and dorsal intersection point of the levator operculi muscle and the opercle; (23) Posterior intersection point between the subopercle and opercle (nlf means neurocranium lateral line foramen in 10, 11, 13 and slf means subopercle lateral line foramen in 16)

A geometric morphometric analysis on all cleared and stained individuals was then performed. The geometric morphometric method is an efficient tool to estimate differences in body shape and head morphology (Kerschbaumer & Sturmbauer, 2011). The use of morphometrics allowed determination of potential performance variation, and applying this to population level variation. A total of 21 landmarks including muscular and skeletal points that capture musculoskeletal shape, muscle size, insertion angles, and lever ratios were marked and then digitized on the right side of the head (Figure 3) contrary to the left side which is more traditionally used. We used the right side of the fish for geometric morphometrics because muscle dissections on the left side of the fish reduced visibility of some landmarks. In digital image acquisition, the landmarks matrix data for each fish image were standardized in position, orientation, and sizes, thus eliminating the effect of these factors from the analysis (DeQuardo et al., 1999). Before entering the shape data into the statistical analysis, nonshape variation was systematically remoted using generalized procustes analysis (GPA) in tpsSuper (Rohlf, 2004). Then images were imported into software tps Dig 2.12 (TPS Software Series; Rohlf, 2006). The resulting coordinates lie in a tangent space, whose variation was calculated to be minimal (Rohlf, 2002) using tps Small (Rohlf, 2003). Therefore transformed landmarks were used in subsequent analyses. The landmark configurations were compared statistically to quantify head shape differences and test for statistical significance of the head shape outlines (DeQuardo et al., 1999).

Three canonical variate analysis (CVA) run in Morpho J 1.02c were performed only (Klingenberg, 2011) to isolate the geometric morphometric shape features that best distinguish the littoral versus pelagic feeding morphology within each species (Foster et al., 2014). Grouping variables were predefined as follows: species (*H. insidiae* or *H. kamiranzovu*), habitat (littoral or pelagic) and sex (female or male). The collection‐location combination variables were also incorporated in the statistical model of Morph J 1.02c. The shape data were quantified through CVA and visualized using deformation grids and drawing outlines from scores along CV1 (Klingenberg, 2011) that represent positive and negative maximum deviations from the mean shape. The two deformation grids and drawing outlines, each representing the mean shape of the ecotype specific were superimposed for comparison of images from littoral and pelagic habitats for each species and for male versus female for each species and for each habitat. This facilitated visualization and inferences of the cranial musculoskeletal shape changes between littoral‐pelagic ecotypes or between sexes and illustrated how they occurred in parallel directions (Colombo et al., 2012; Muschick et al., 2012). The drawing outlines and deformation grids were also performed for the same reason. The advantage of the drawing outlines is its clarity since the semi‐landmarks were collected. The curvatures of targeted anatomical structures showing variation were illustrated entirely in two dimensions. By drawing fully‐formed lines to connect all the landmarks, it gives the information reflected in the data.

### 2.3 Bite model

**FIGURE 4:**
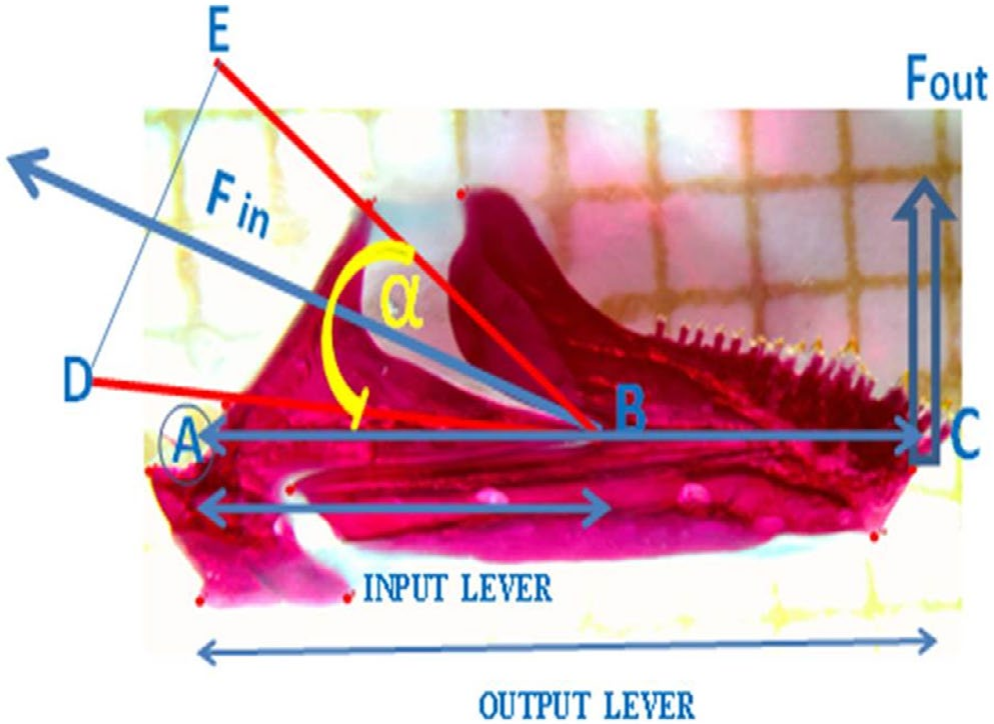
Bite force model illustration. *F*_out_ is a Force output at the tip of the lower jaw. It depends on the muscular in put force, α is the angle of the insertion of the muscle onto the jaw, and the ratio of in lever arm (*L_i_*) to out lever arm (*L_o_*). The angle of insertion of the jaw muscles changes during jaw closing. Muscles have a low angle (α) relative to the in lever when the jaw is open and during contraction, the angle increases until the jaw is completely closed and output force becomes maximal. The point A is the lower jaw joint. The distance AC is the output lever. The distance AB is input lever. *F*_in_ is input force. Arrow BF_in_ is the action direction of *F*_in_ that is generated by the mandibular muscles contraction. The triangle DBE represents the expansion of *F*_in_—input force of the muscle in action during contraction. E and D are determined by the extension of the action direction of the A2 division of the adductor mandibular (in contraction) from its anterior most tip‐point and the most antero‐ventral points attached of the dentary bone of the lower jaw

The jaws were first modeled as simple levers as proposed in a number of studies of cichlids and other fishes (Barel, 1983; Herrel, McBrayer et al., 2010; Herrel, Moore et al., 2010; Holzman et al., 2012). The distance from the mid‐point of the articular quadrate joint to the mid‐point of the interopercle‐angular joint was used as the in‐lever for jaw opening. The jaw closing in‐lever, *L_i_*, was measured as the distance between the mid‐point of the articular quadrate joint and the insertion site of the lower jaw adductor muscle. The out‐lever for both jaw opening and jaw closing, *L_o_*, was measured as the distance between the mid‐point of the articular quadrate joint and the tip of the anterior most tooth (Figure 4). When a fish catches its prey, muscle forces during biting are transmitted through the lower jaw closing lever system. Therefore, we inferred the maximum force produced during contraction of the jaw muscles. Muscle cross sectional area of all the three muscles A1, A2 and A3 were measured after immersion of the muscles in 30% nitric acid (Herrel et al., 1998). The muscle fibers were teased apart (after 48–50 hr) and photographed to digitally obtain the average fiber length. Then thirty individual fibers per A1, A2, and A3 pinnate muscles were measured using Image J (Collins, 2007). Physiological Cross Section Area (PCSA) was estimated by dividing the muscle volume by mean fiber length (Tkint et al., 2012). The muscle density was supposed to be 1 g × cm^−3^ (Westneat, 2003) and the unit contraction force was assumed to be 19 N/cm^2^ (Akster et al., 1985). The contraction inferred bite force was calculated following the formula: (*F*_in_ = PCSA * 19 N/cm^2^). The closing force exerted from the tip of the jaw was calculated according to the following formula: *F*_out_ = *F*_in_ * (*L*_i_/*L*_o_) * sin*α* (where *α* is an insertion angle). Thirty fibers per muscle were measured for length. Weight of the three muscles types, A1, A2 and A3 pinnate muscles were also measured and later used for physiological cross section area (PCSA) quantification instead of anatomical cross sectional area.

### 2.4 Kinematic transmission

When the fish protrudes its jaws to capture and swallow prey, movements are transmitted through four skeletal elements that can be modeled as a four‐bar linkage (Hulsey & Garcia de León, 2005; Westneat, 1995a, 1995b). The bones that make up this linkage system are the nasal bone, the lower jaw, the maxilla and the suspensorium. In cichlids, this morphological elements correspond to mechanical elements in the system: the suspensorium acts as the fixed link, the nasal functions as the coupler, the maxilla serves as the output link and the coronoid portion of the lower jaw acts as an input link (Hulsey & Garcia de León, 2005; Tkint et al., 2012). When the fish open and close the mouth, the maxilla rotates in response to lower jaw depression. The kinematic transmission (KT) of motion of this system can be defined as a ratio between the output rotation of the maxilla and input rotation of the lower jaw Tkint et al., 2012), following the formula:

$$\text{KT}=\frac{L_{\text{output}}}{L_{\text{input}}}.$$

All angles of the link were defined: the initial angle in relation between the lower jaw and the fixed link or the starting angle. For each specimen, a starting angle of 15° had been quantified repeatedly as the diagonal distance (*E*) from the place where the nasal bone is fixed on the maxilla at the site where the link of the lower jaw meets the fixed link to the coronoid process. The diagonal isolating the connection into two triangles was established. This allowed to accurately quantifying all the angular relationships between the links, including the starting angle, from the cosine formula:

$$\text{Cos}\left(\text{angle}\right)=\frac{\left(A^{2}+B^{2}‐E^{2}\right)}{\left(2AB\right)}.$$

An input angle of 30° was decided as a suitable rotation of the lower jaw, although there is need to study the amount of the lower jaw rotation in Lake Kivu haplochromines. Using joints coordinates of the linkage on the dissection images, we determined the size of different links, the starting angle and the input angle. The distance between two landmarks of the targeted anatomical structures or their midpoints (Figures 3 and 4) were calculated using the formula to find the squared distance between two landmarks; *d* ^2^ = *X* ^2^ + *Y* ^2^ where *X* is the positive difference between the *x*‐coordinates, the *x*‐coordinates are the first numbers in each set of coordinates and *Y* is the positive difference between the *y*‐coordinates the *y*‐coordinates are the second numbers in each set of coordinates. The actual distance between two points (*d*) is the square root of *d* ^2^. To calculate the midpoints of the line segments mentioned, we considered the formula cited above taking into account that the midpoint of the line segment has the coordinates: ((*x*_1_ + *x*_2_)/2, (*y*1 + *y*2)/2)). Then, calculation of the distance from one extreme point of the targeted anatomical structure to its corresponding midpoint, we used the formula of *d* ^2^ (mentioned above). Then, all the dissection images with their landmarks and midpoints coordinates were implemented in Excel R (Microsoft Corporation) (Tkint et al., 2012). The conversion from dpi to real distance from images gave the same results. We quantified the mechanical attributes of each linkage by the kinematic transmission (KT) as per Muller (1987) and Hulsey and Garcia de León (2005). The four‐bar linkage allowed calculating the angular rotation of the output link. Then, we determined the maxillary KT by dividing the output rotation by the input rotation of 30°. Its numerical output from calculation was used in the comparisons of pelagic to littoral fish and of male to female fish.

The kinematic efficiency (KE) as a measure of suction feeding of a fish was quantified by dividing the outlever by the inlever for jaw opening (Tkint et al., 2012). It indicates the speed at which a fish can open its mouth.

During data recording, the averages were calculated per species, per habitat and per sex for the following 15 variables for whole individuals of fish population in each sampling location: mass of A1, A2, and A3 muscles, fiber length of A2 and A3, the head length, the ratio between ascending arm of the premaxillary and the head length, the total force production of A2 and A3, the kinematic transmission coefficient of the anterior jaw four‐bar linkage, the mouth opening lever ratio, the mouth closing lever ratio and the angle between the ascending arm of the premaxillary as well as the dentigerous area of the dentary were calculated. These variables were used in the formula of calculation of total bite force, kinematic efficiency and kinematic transmission for comparison of the littoral and pelagic.

### 2.5 Statistical analysis

Three canonical variate analyses relating shape with habitat (littoral versus pelagic), sex (female and male) and at convergency level were performed to define which canonical axis most explains the difference between habitats and sex. Finally, to find if littoral versus pelagic and female versus male divergences could occur along similar direction within littoral and within pelagic, within *H. kamiranzovu* and *H. insidiae*.

To reduce data dimensionality of geometric morphometrics of the shape data set, a principal component analysis (PCA) was initially used to examine patterns of morphological variation for both species in relation to the habitat and sex types. Since the assumption of the null hypothesis was defined that the musculoskeletal shape of the studied fish species haplochromines are not different. The test for normality on the PCA loadings showed that body and the skull shape variations in both species were not normally distributed (*p* = .126); therefore, the shape data were subjected to a nonparametric multivariate analysis of variance (npMANOVA) using PAST (Hammer et al., 2001). This npMANOVA was used to test for significant differences in the distribution of habitat types (littoral versus pelagic) and sex (male versus females) for all populations in morphospace using a permutation procedure for Procrustes distances that calculate means among groups (cited above) in order to establish the distance benchmark (Anderson, 2001). The habitat, sex types and shape being independents and dependent variables, respectively.

The significant differences between ecotypes/sexes do tell us whether they are different, and how they are different with *p*‐values. The collection location was included in the statistical model.

The npMANOVA is an equivalent design to an ANOVA that allowed also testing fifteen biomechanical variables cited above and their interactions. Differences of feeding performance between species and sex in relation to habitat were analyzed with a glm (generalized linear model) implementation of a two‐way ANOVA with inclusion of Head length (HL) as covariate. All statistical analyses were run using SAS 9.2. (SAS Institute Inc.2013. SAS® 9.4 Statements: Reference: SAS Institute Inc.)

## 3 RESULTS

### 3.1 Habitat‐related musculoskeletal shapes differences

**FIGURE 5:**
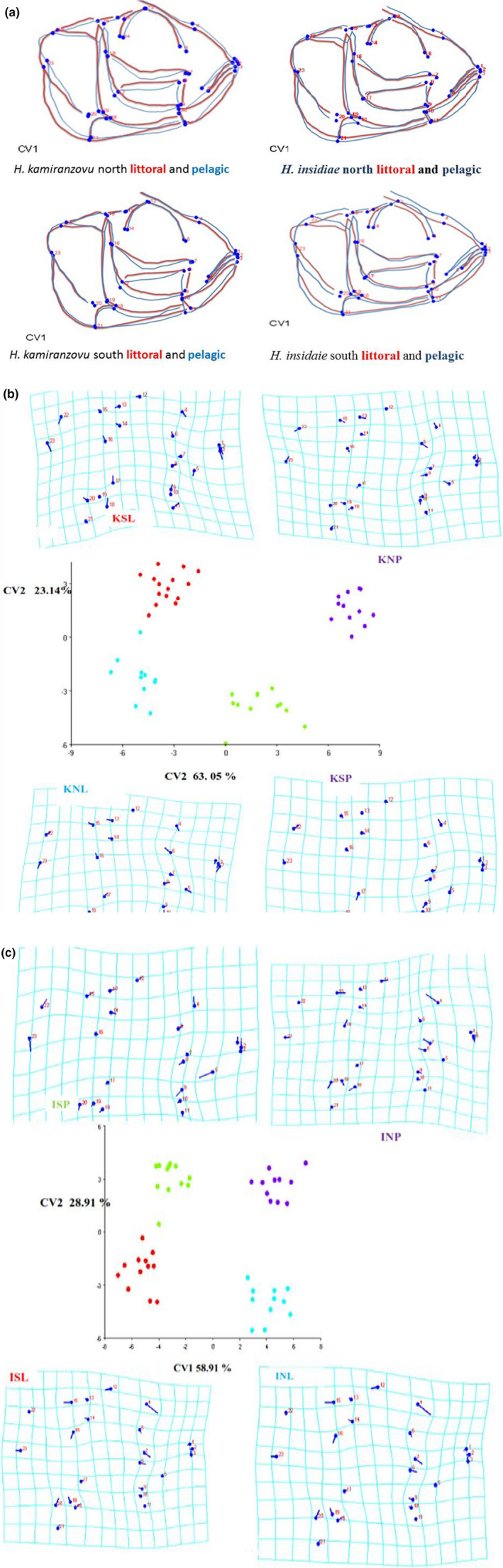
(a) Consensus configuration of the cranio musculoskeletal shape of the littoral versus pelagic of both haplochromis fish species caught from littoral and pelagic Lake Kivu habitat. Landmarks 16 and 7 seem to be nearly identical for the two populations of *Haplochromis kamiranzovu* south littoral and pelagic fish. However, those lines connecting them allowed visualizing the significant shape difference between the positions of their A_1_ mandibular as well as its position in the northern part for the same fish population. (b) CV1 versus CV2 plot habitat‐related musculoskeletal shapes differences within *H. kamiranzovu* and their deformation grids. Legends used: KNL: *H. kamiranzovu* north littoral; KSP: *H. kamiranzovu* south pelagic; KNP: *H. kamiranzovu* north pelagic; KSL: *H. kamiranzovu* south littoral. (c) CV1 versus CV2 plot habitat‐related musculoskeletal shapes differences within *Haplochromis insidiae* and their deformation grids. Legends used: ISP: *H. insidiae* south pelagic; ISL: *H. insidiae* south littoral; INP: *H. insidiae* north pelagic; INL: *H. insidiae* north littoral

The musculoskeletal shapes of the pelagic versus littoral differed significantly within the two haplochromine fish species. Individuals of *H. insidiae* and *H. kamiranzovu* have an ascending arm of the premaxilla positioned more dorsally (landmark 4), a larger preorbital region of the skull (landmarks 4 to 11) and an A2 shifted more posteriorly (landmark 17) in pelagic habitats (Figure 5a–c).

These assertions hold true in both species. A pairwise comparison npMANOVA performed between pelagic versus littoral specimens within *H. insidiae* and *H. kamiranzovu* species revealed that these groups were significantly different in head shape (*p* = .001 and *p* = .026), respectively. The results of Wilks' Lambda test and Pillai trace test were 0.07 and 1.38, respectively. The degrees of freedom and *F* values of the above tests were Df1 = 11; Df2 = 147; the *F* = 45.99 and Df1 = 11; Df2 = 149 and *F* = 33, respectively. The plot of the canonical variate analyses relating shape with habitat (littoral versus pelagic) defined the first and the second canonical axis that most explains the difference between habitats. The CV1 versus CV2 explained 63.05% and 23.14% of variations, respectively in *H. kamiranzovu* (Figure 5b). The CV1 versus CV2 explained 58.91% and 28.87% of variations, respectively in *H. insidiae* (Figure 5c). Findings show that littoral versus pelagic divergence occurred along similar direction within littoral and within pelagic, within *H. kamiranzovu* and *H. insidiae*.

Kinematic transmission (KT) reported in Table 2 has large standard errors, meaning that any pairs of groups are not statistically different from each other. There is statistically nonsignificant but consistent trend that littoral groups have smaller KT value than comparable pelagic groups. *H. insidiae* sampled in northern littoral (INL) and *H. insidiae* sampled in northern pelagic (INP) comparison was *p* = .001 and the rest of the comparisons were *p* < .05 as shown by CVA.

**TABLE 2:** Quantification of feeding performance‐species habitat‐related sex

OUT’s	Inferred total bite force (*N*)	KE^a^	KT^a^
(*n*)	Mean ± *SE*	Mean ± *SE*	Mean ± *SE*
*Haplochromis insidiae* pelagic male (11)	0.3 ± 0.1	6.2 ± 1.6	0.7 ± 0.9
*H. insidiae* littoral male (10)	0.5 ± 0.2	6.4 ± 1.4	0.8 ± 0.7
*H. insidiae* pelagic female (13)	0.3 ± 0.2	6.1 ± 0.7	0.8 ± 0.9
*H. insidiae* littoral female (11)	0.2 ± 0.1	4.9 ± 1.3	0.9 ± 0.8
*Haplochromis kamiranzovu* pelagic male (10)	0.3 ± 0.2	6.9 ± 0.9	0.7 ± 0.6
*H. kamiranzovu* littoral male (13)	0.4 ± 0.2	6.2 ± 0.6	0.8 ± 0.5
*H. kamiranzovu* pelagic female (14)	0.3 ± 0.2	5.9 ± 0.6	0.6 ± 0.7
*H. kamiranzovu* littoral female (13)	0.3 ± 0.1	5.5 ± 0.8	0.8 ± 0.8
Main effect‐species			
*F*‐values F1, 87	2.77	0.61	2.2
*p*‐Values	.01	.44	.16
Main effect‐sex			
*F*‐values F1, 87	2.15	8.65	0.49
*p*‐Values	.15	.004	.49
Main effect‐habitat			
*F*‐values F1, 87	2.55	0.87	3.4
*p*‐Values	.17	.05	.25

Note

Inferred total bite force and kinematic transmission (KT) are variables that indicate the ability of a fish to generate strong feeding events, but they are not actual measurements of performance in living fishes.

The means and their standard deviations for the 30 fiber measurements for estimating mean length for each muscles A1; A2 and A3 in *Haplochromis insidiae* were 0.018 ± 0.004; 13.22 ± 3.29; 6.53 ± 3.02 and in *Haplochromis kamiranzovu* were 0.020 ± 0.004; 14.99 ± 3.43; 6.19 ± 0.99, respectively.

*F*‐values were approximated using Wilk's lambda. The statistical power associated with using MANCOVA with bite force, kinematic transmission and kinematic efficiency coefficients data, our model results on effect strengths by use of *p*‐values. When testing for species, sex and habitat via MANCOVA, all terms had no significant difference effects on bite force, kinematic transmission and kinematic efficiency coefficients variations in each species.

Abbreviations: *N*, inferred total bite force in Newton; *n*, number of specimens examined.

^a^ Unit less.

### 3.2 Sex‐related musculoskeletal shapes differences

**FIGURE 6:**
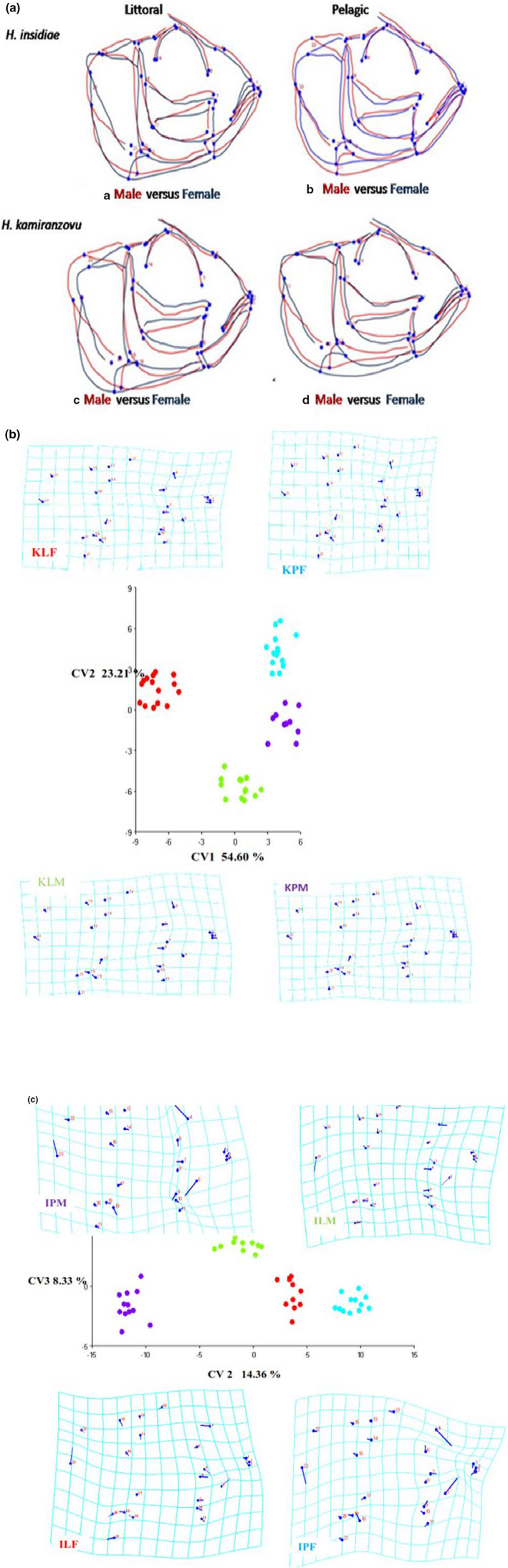
(a) Consensus configuration of the male versus female of the facial musculoskeletal shape of both studied haplochromine fish species caught in Lake Kivu. Colors of acronyms correspond to colors dots used in the CVA plots and deformation grids. (b) Consensus configuration of the male versus female of the facial musculoskeletal shape of *Haplochromis kamiranzovu* fish species caught in Lake Kivu. Legends used: KLF: *H. kamiranzovu* littoral female; KLM: *H. kamiranzovu* littoral male; KPF: *H. kamiranzovu* pelagic female; KPM: *H. kamiranzovu* pelagic male. (c) Consensus configuration the male versus female of the facial musculoskeletal shape of *Haplochromis insidiae* fish species caught in Lake Kivu. Legends used: ILF, *H. insidiae* littoral female; ILM, *H. insidiae* littoral male; IPF, *H. insidiae* pelagic female; IPM, *H. insidiae* pelagic male

The musculoskeletal shapes of the male versus female differed significantly in the two haplochromine fish species **(**Figure 6a–c).

The male individuals of both haplochromines species have a longer head while the females have a shorter head across the Lake Kivu (landmarks 1–22).

The female individuals in both haplochromines species have a ventral higher larger buccal cavity across the Lake Kivu than males (landmarks 1; 16 and 21). A pairwise comparison npMANOVA performed between female versus male specimens within *H. insidiae* and *H. kamiranzovu* species from across Lake Kivu revealed that sexes were significantly different in CVA musculoskeletal shape (*p* = .019 and *p* = .030 respectively).

The musculoskeletal shape in littoral individuals was consistent with smaller mean of kinematic transmission in both sexes of the both haplochromines species and with none significant differences (*p* = .25; Table 2).

### 3.3 Convergence phenotypes in both haplochromines species

**FIGURE 7:**
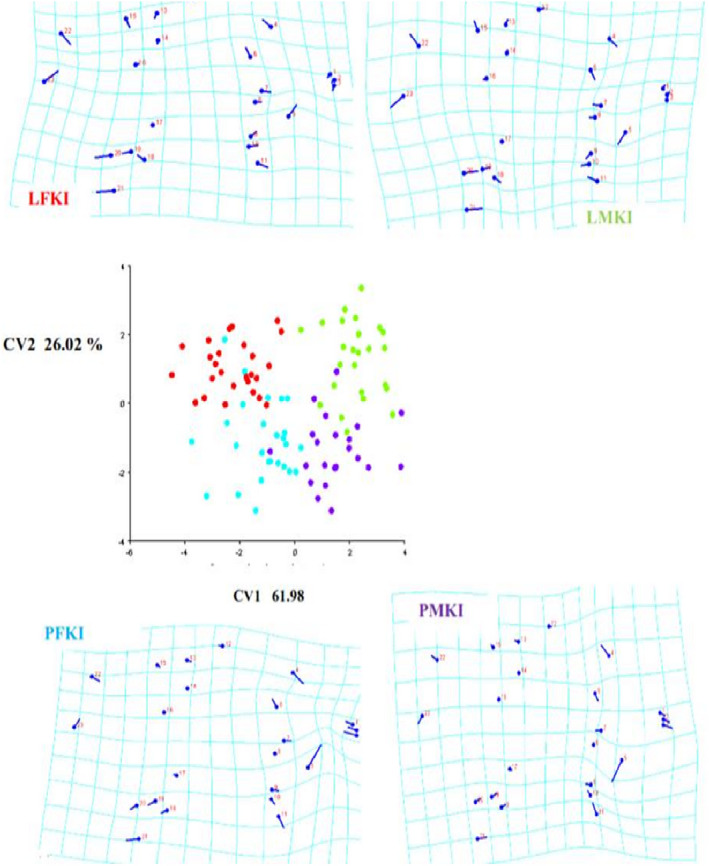
Convergence phenotypes in both haplochromines species: male individuals have longer heads than females. Pelagic females have a A2 muscle shifted posteriorly. LFKI: Littoral female individuals of *Haplochromis kamiranzovu* and *Haplochromis insidiae*. LMKI: Littoral male individuals of *H. kamiranzovu* and *H. insidiae*. PFKI, pelagic female individuals of *H. kamiranzovu* and *H. insidiae*; PMKI: pelagic male individuals of *H. kamiranzovu* and *H. insidiae*

Similar sex‐related phenotypes of *H. insidiae* and *H. kamiranzovu* also appear to reflect convergent musculoskeletal shape that is associated with littoral versus pelagic habitats. The males of *H. insidiae* and *H. kamiranzovu* have longer heads than their respective females respectively (Figure 7). Generally, pelagic female individuals in both species had a mandibulae A2 muscle shifted posteriorly (Figure 7). This change in the A2 muscle was inferred to be associated with a higher mean total bite force and a higher mean kinematic transmission. The inferred total bite forces for females of both species were higher in the pelagic zone with no significant difference (*p* = .25; Table 2).

## 4 DISCUSSION

### 4.1 Habitat‐related musculoskeletal shapes differences

The trophic morphology of cichlids generally changes in predictable ways when species diverge along the littoral versus pelagic axis (Bouton et al., 1998; Cooper et al., 2010; Parsons et al., 2015; Wainwright & Richard, 1995). Morphological variation in the preorbital region of the skull of haplochromines also commonly reflects substantial differences in biomechanics of fish feeding (Cooper et al., 2010; Cooper & Westneat, 2009; Parsons et al., 2011; Westneat, 1995a, 1995b, 2003). Pelagic fish generally feed on evasive prey (Yaniv et al., 2014) while littoral fish feed mostly on nonevasive or attached prey they remove from the substrate (Thomaz & Cunha, 2010). Pelagic individuals in both species studied here displayed a larger preorbital region. This was accompanied by the more dorsally position of the ascending arm of the premaxillary bone. This bone itself is important to influence the protrusion of the upper jaw during suction feeding (Staab et al., 2011). This seems to suggest that the pelagic individuals were better suited to jaw protrusion and therefore suction feeding. This seems to agree with the cichlid results that shows that more pelagic species like *Metriaclima zebra* have morphologies that would be expected to produce better suction feeding (Albertson & Kocher, 2001; Cooper et al., 2010). This result is supported with direct experimental evidence by another paper from the same group (Matthews & Albertson, 2017). These cichlid comparisons are suction versus. biting species where in littoral lineages possess morphological traits as being more benthic and therefore better at biting compared to pelagic (Albertson & Kocher, 2001; Cooper et al., 2010). About the variability in food types in the littoral environment versus pelagic one, findings showed that both haplochromines species caught in pelagic zones contained consistently greater frequencies of copepod, cladocera, *Planktolyngbia undulata*, *Microcystis* sp. than in littoral zones. The occurrence of zooplankton in *H. kamiranzovu* stomachs was significantly higher in the pelagic than the littoral zone (*p* < .05), while the opposite pattern existed for *H. insidiae*. In overall, the planktons occurred significantly higher (*p* < .05) in the pelagic stomach of the females while the opposite trend existed in both haplochromine males across Lake Kivu (Munyandamutsa & Agbebi, 2015).These results are also consistent with findings in the New World cichlid *Amphilophus citrinellus* that when found in different habitats have independently evolved parallel changes related to craniofacial shape (Barluenga & Meyer, 2004, 2010; Elmer et al., 2010). The preorbital size difference among these paired habitat lake suggests the occurrence of great rapidity of adaptation when fishes invade new habitats lake with numerous vacant niches (Cooper et al., 2010). The capability to quickly evolve jaws and preorbital of different sizes and shift of jaw muscles were associated to differences in littoral and pelagic feeding modes in cichlids and in the marine damselfishes (Azuma et al., 2008; Cooper et al., 2009). The expansion of the preorbital region of the skull for both pelagic haplochromines led us to predict that this enlargement would allow them to process large prey in pelagic habitat as observed to Lake Malawi cichlids (Le Pabic et al., 2016).

This morphological divergence is an evidence of local adaptation, implying reproductive isolation and genetic divergence. However, we did not investigate if these populations are isolated or not. However, these findings most likely result from morphological plasticity in response to different mechanical feeding regimes (Gunter et al., 2013; Parsons et al., 2015). The adaptive phenotypic plasticity is a capability of an organism to cope local environments. This trait is common to East African cichlids and increasingly contributing to evolution (Gunter et al., 2013). For instance, *Astatoreochromis alluaudi* displayed adaptive phenotypic plasticity in its pharyngeal jaw apparatus in response to different diets, the pharyngeal jaws modified their size, shape and dentition. Hard food items induced robust molariform tooth shape with short jaws and strong internal bone structures, while soft diet induced a gracile papilliform tooth morphology with elongated jaws and slender internal bone structures.

The main differences between the pelagic and littoral populations in both species, and in both habitats are shown by the geometrics morphometrics results. These derived from the dorsal shift of preorbital landmarks. The overall cranial morphology of both species studied also showed that the littoral specimens have shorter heads; shorter jaws, nonexpanded opercula bones and the eyes positioned more dorsally which is in accordance with habitat divergence in other cichlids (Albertson & Kocher, 2001; Barel, 1983; Otten, 1983). The shortening of the jaws and the dorsal shift of the eye have been reported to increase the mechanical advantage of “biter” fish (Albertson & Kocher, 2001; Tkint et al., 2012) as this putatively allows the jaw muscles to expand during jaw closure. It is likely having room for a bigger muscle, is a more obvious reason than allowing the muscle to expand during use. This is more compelling as an explanation for the observed trend.

The observed decrease in the KT values of the anterior jaw mechanics in both species in the littoral habitats suggested that there could be more biting feeding in littoral zones during mouth closing (Hulsey & Garcia de León, 2005; McGee et al., 2013). Similar patterns in trophic morphologies have been found in Lakes Malawi, Tanganyika, and Victoria (Meyer, 1989; Meyer et al., 1993; Muschick et al., 2012).

### 4.2 Sex‐related musculoskeletal shapes differences and feeding performance

Sexual differences in trophic morphology of the two Lake Kivu haplochromine species were also recovered. The finding is that male individuals of *H. insidiae* and *H. kamiranzovu* are larger than females in Lake Kivu. This could be related to territorial defense (Erlandsson & Ribbink, 1997; Hudman & Gotelli, 2007; Passos et al., 2013; Ptacek & Travis, 1997; Schütz & Taborsky, 2011; Tsuboi et al., 2012). Sexual dimorphism in cichlid fishes is common and in species such as *Lamprologus callipterus* and *Cichlasoma dimerus* males are often larger than females (Alonso et al., 2011; Hulsey et al., 2015; Ota et al., 2010; Schîutz et al., 2006). There are many potential reasons for this. Females of many species tend to grow more slowly than males once they reach adulthood due to increased energetic efforts in producing eggs during reproduction (Shine, 1989). Another reason for size differences between the sexes is sexual selection. For example, in the monomorphic Midas cichlid (*Amphilophus citrinellum*), females choose large aggressive males that might better defend territories (Barlow, 1998). Male size might also provide a reliable signal of territory quality and, females may profit from shelter and food provided by the territory (Hermann et al., 2015). Sex also appears to influence shape differences in the cranial morphology of the Lake Kivu cichlids.

### 4.3 Convergence phenotypes in both haplochromines species

The head of both species from Lake Kivu is commonly longer in male individuals. These differences are similar to those reported in the genus *Tropheus* of Lake Tanganyika in which shape variation between populations and between sexes in *Tropheus moorii* and *Tropheus polli* was primarily located in the cranial region (Herler et al., 2010). This type of parallel patterns in sexual differentiation between two closely related species also is similar to findings in other vertebrate taxa (such as lacertid lizards that show sexual dimorphism with male individuals having longer heads than conspecific females (Harmon et al., 2005; Žagar et al., 2012). The larger buccal cavity observed in both female haplochromine fish species was explained by Herler et al. (2010) and Cooper et al. (2011) who proposed that larger buccal cavity enables female to adapt to mouthbrooding. The apparent divergence in trophic phenotypes across both habitats and the sexes feeding morphology in *H. insidiae* and *H. kamiranzovu* is likely to be influenced by both natural and sexual selection. Sexual dimorphism implies also that there was sexual selection acting on the trait. Similar studies involving other types of the Lake Kivu fishes are recommended to test the evidence of the above trophic patterns observed and their genetic basis.

## CONFLICT OF INTEREST

The authors have no conflict of interest to declare.

## AUTHOR CONTRIBUTION

**Philippe Sanzira Munyandamutsa:** Conceptualization (lead); Data curation (lead); Formal analysis (lead); Funding acquisition (lead); Investigation (lead); Methodology (lead); Project administration (lead); Resources (lead); Software (lead); Supervision (equal); Validation (equal); Visualization (lead); Writing‐original draft (lead); Writing‐review & editing (lead). **Wilson Lazaro Jere:** Conceptualization (lead); Data curation (lead); Formal analysis (lead); Funding acquisition (supporting); Investigation (lead); Methodology (lead); Project administration (lead). **Daud Kassam:** Conceptualization (lead); Data curation (equal); Formal analysis (lead); Funding acquisition (lead); Investigation (lead); Methodology (lead); Project administration (lead); Resources (lead); Software (lead); Supervision (lead); Validation (lead); Visualization (equal); Writing‐original draft (supporting); Writing‐review & editing (supporting). **Austin Mtethiwa:** Conceptualization (lead); Data curation (supporting); Formal analysis (supporting); Funding acquisition (lead); Investigation (supporting); Methodology (equal); Project administration (lead); Resources (equal); Software (equal); Supervision (lead); Validation (lead); Visualization (equal); Writing‐original draft (supporting); Writing‐review & editing (equal).

## Data Availability

Sampling locations and fish morphological data will be archived in dryad https://doi.org/10.5521/dryad.12311.
